# Influence of gonadectomy and age at gonadectomy on the risk of developing intervertebral disc herniation in French Bulldogs: a retrospective study

**DOI:** 10.3389/fvets.2025.1729833

**Published:** 2025-12-05

**Authors:** Marco Tabbì, Alessandro Troisi, Domenico Fugazzotto, Romina Marcoccia, Giorgia Pettina, Simone Minniti, Girolamo Messina, Temy Coppola, Gaetano Principato, Federica Arrigo, Giuseppe Barillaro, Daniele Troiano, Angela Polisca, Viola Zappone

**Affiliations:** 1 Department of Veterinary Sciences, University of Messina, Messina, Italy; 2 School of Biosciences and Veterinary Medicine, University of Camerino, Macerata, Italy; 3 Ospedale Veterinario San Francesco, Castagnole, Italy; 4 Clinica Veterinaria San Giorgio, Reggio Calabria, Italy; 5 Department of Veterinary Sciences, University of Perugia, Perugia, Italy; 6 Centro Traumatologico Ortopedico Veterinario, Arenzano, Italy; 7 Clinica Veterinaria Etiopia, Roma, Italy

**Keywords:** intervertebral disc herniation, IVDH, gonadal hormones, gonadectomy, age at gonadectomy, dog, French Bulldog

## Abstract

The French Bulldog has become increasingly popular worldwide, but selective breeding for brachycephalic traits has increased its predisposition to several neurological disorders. Among these, intervertebral disc herniation (IVDH) is one of the most common causes of spinal cord compression, pain, and neurological deficits. Although genetic predisposition plays a key role, previous evidence suggests that gonadal hormones factors may also adversely affect skeletal development and spinal biomechanics, thus influencing risk of IVDH. The aim of this study is to evaluate the influence of gonadectomy and age at gonadectomy on the risk of developing IVDH in French Bulldogs. Medical records of 2,101 French Bulldogs were retrospectively reviewed. Dogs with complete clinical and reproductive histories were included and classified into two groups: affected (A group, with IVDH) and control (C group, without IVDH). Early-gonadectomized dogs (<12 months) showed a significantly higher risk of IVDH compared with intact dogs. Early-gonadectomized males were nearly twice as likely to develop IVDH (OR = 1.92) than intact males, while early-gonadectomized females exhibited an even stronger association (OR = 9.84) compared with late-gonadectomized (OR = 2.77) and intact females. The preliminary results of this study suggest a potential influence of gonadectomy and age at gonadectomy on the risk of developing IVDH in French Bulldog. Therefore, the age at which gonadectomy is performed should be carefully considered in this and other predisposed chondrodystrophic breeds. These findings highlight the importance of gonadal hormones and the need for further research to evaluate the differential effects of early age gonadectomy.

## Introduction

The French Bulldog is a canine breed that has grown in popularity worldwide over the last two decades (1–5). Intensive morphological selection aimed at enhancing brachycephalic traits through selective breeding has markedly increased the French Bulldog’s predisposition to a wide range of disorders, particularly neurological conditions. Consequently, a high prevalence of both intracranial and spinal diseases has been reported in this breed, including intervertebral disc disease (IVDD) (6–10).

Intervertebral disc herniation (IVDH) is defined as the pathological displacement of an intervertebral disc into the vertebral canal through a ruptured annulus fibrosus. It is the most prevalent IVDD and one of the main causes of pain and neurological dysfunction in dogs (11–13). The progressive degeneration seen in IVDH is an aging process characterized by metaplasia, dehydration and calcification of the *nucleus pulposus* (NP). Biomechanical strain and trauma accelerate these changes that ultimately result in herniation of degenerative NP (12–16). In chondrodystrophic breeds such as French Bulldogs and Dachshunds, IVDH commonly occurs in an acute or peracute form, according to Hansen’s type I disc disease. This involves extrusion of the NP through a torn annulus fibrosus, which subsequently compresses the vertebral canal. However, disc protrusions, classified as Hansen’s type II disease, can also occur. This is characterized by gradual displacement of the NP within the damaged annulus fibrosus and progressive bulging toward the vertebral canal (17, 18). Neurological damage resulting from IVDH is due to compression, contusion, hemorrhage and laceration of the spinal cord (18, 19). The clinical signs vary depending on the spinal segment affected and the degree of injury (20).

In French Bulldogs, IVDH is one of the most frequently diagnosed causes of myelopathy. A single-center retrospective study of 343 French Bulldogs referred to a neurological reference center between 2002 and 2016 found that IVDH was responsible for 70.3% of all myelopathies, 45.5% of all neurological disorders, and 5.5% of all reasons for clinical consultation in the breed. The anatomical distribution of IVDH showed a higher prevalence of thoracolumbar and lumbar locations (60.2%) than cervical locations (39.8%) while the onset typically occurred in adulthood and was significantly associated with age over 3 years (1). In another retrospective study of 80 French Bulldogs referred to a Veterinary Hospital for IVDH the prevalence of lumbosacral localization was 91.3% (21).

The possible role of gonadectomy as a risk factor for IVDH is a topic of growing interest. While evidence in French Bulldogs remains limited, studies in other chondrodystrophic breeds, particularly Dachshunds, have shown that early gonadectomy can alter skeletal development and lead to an increased incidence of orthopedic and neurological disorders, including IVDH (22–26). It is hypothesized that the hormonal changes resulting from ovariectomy or orchiectomy, especially when performed before skeletal development is complete, interfere with vertebral growth and spinal biomechanics, thereby promoting degenerative processes (22, 26). Gonadectomy is a widespread practice in the pet population for reasons of management and reproductive welfare. Therefore, it is essential to clarify the association between reproductive status and the risk of IVDH in predisposed breeds, such as French Bulldogs.

The aim of this study is to evaluate the influence of gonadectomy and age at gonadectomy (less than 12 months vs. more than 12 months) on the risk of developing IVDH in French Bulldogs. Based on previous evidence linking gonadal hormones to skeletal development, the authors hypothesized that gonadectomy increases the risk of IVDH in French Bulldogs, particularly when performed before 12 months of age.

## Materials and methods

The medical records of French Bulldogs referred to various Italian Veterinary facilities between January 2019 and March 2025 were retrospectively reviewed. Only subjects with complete clinical and reproductive histories were included in the study. Signalment (age, sex, and weight) and complete reproductive history were collected for each patient. The enrolled subjects were first divided into an affected group (group A) and a control group (group C) based on the presence or absence of IVDH, respectively. Group A included 601 subjects, 343 males and 258 females. Group C included 1,500 subject, 803 males and 697 females. Then, in both groups, subjects were further classified according to their reproductive status as intact (IN), early gonadectomized (EG—gonadectomized before 12 months of age) or late gonadectomized (LG—gonadectomized after 12 months of age).

The diagnosis of IVDH was performed with complete neurological examinations and magnetic resonance imaging (MRI). Informed consent was obtained from all owners prior to diagnostic procedures. The MRI protocol was performed in accordance with the guidelines proposed by the Canine Spinal Cord Injury Consortium (CANSORT-SCI) for canine IVDH (27). Each patient was positioned in dorsal recumbency with non-magnetic foam positioning aids to achieve a straight spinal alignment. MRI was performed in transverse, sagittal, and dorsal planes using T2-weighted (T2W), T1-weighted (T1W), and short T1 inversion recovery (STIR) sequences. All MRIs were interpreted by a European College of Veterinary Neurology (ECVN) Resident under the direct supervision of an ECVN Diplomate.

The collected data was analyzed using descriptive statistics. The Shapiro–Wilk test was used to verify data distribution and assess normality, while the chi-square test was used to examine differences between groups. This analysis enabled the calculation of the odds ratio (OR) with a 95% confidence interval (CI) to determine the association between gonadectomy and the presence of IVDH. A *p*-value of less than 0.05 was considered statistically significant. The statistical analyses were performed using software Prism v. 5.01 (Graphpad Software Ltd., United States, 2007).

## Results

A total of 2,101 French Bulldogs were analyzed during the study period. Of these, 601 were diagnosed with IVDH (A group), while the remaining 1,500 were not affected (C group). The age and weight of subjects affected by IVDH, divided by sex and experimental group, are shown in Table 1.

**Table 1:** Age (years, mean ± SD) and body weight (kg, mean ± SD) of French Bulldogs affected by IVDH, divided by sex and experimental group: IN, intact; EG, early gonadectomized; LG, late gonadectomized.

Gender	Group	Age	Weight
Male	IN	6.06 ± 3.44	12.04 ± 2.19
	EG	6.61 ± 3.55	12.05 ± 2.45
	LG	5.61 ± 3.82	11.27 ± 2.44
Female	IN	5.64 ± 3.4	10.69 ± 1.85
	EG	6.11 ± 3.18	11.07 ± 1.74
	LG	5.84 ± 2.8	11.08 ± 1.77

**Figure 1:**
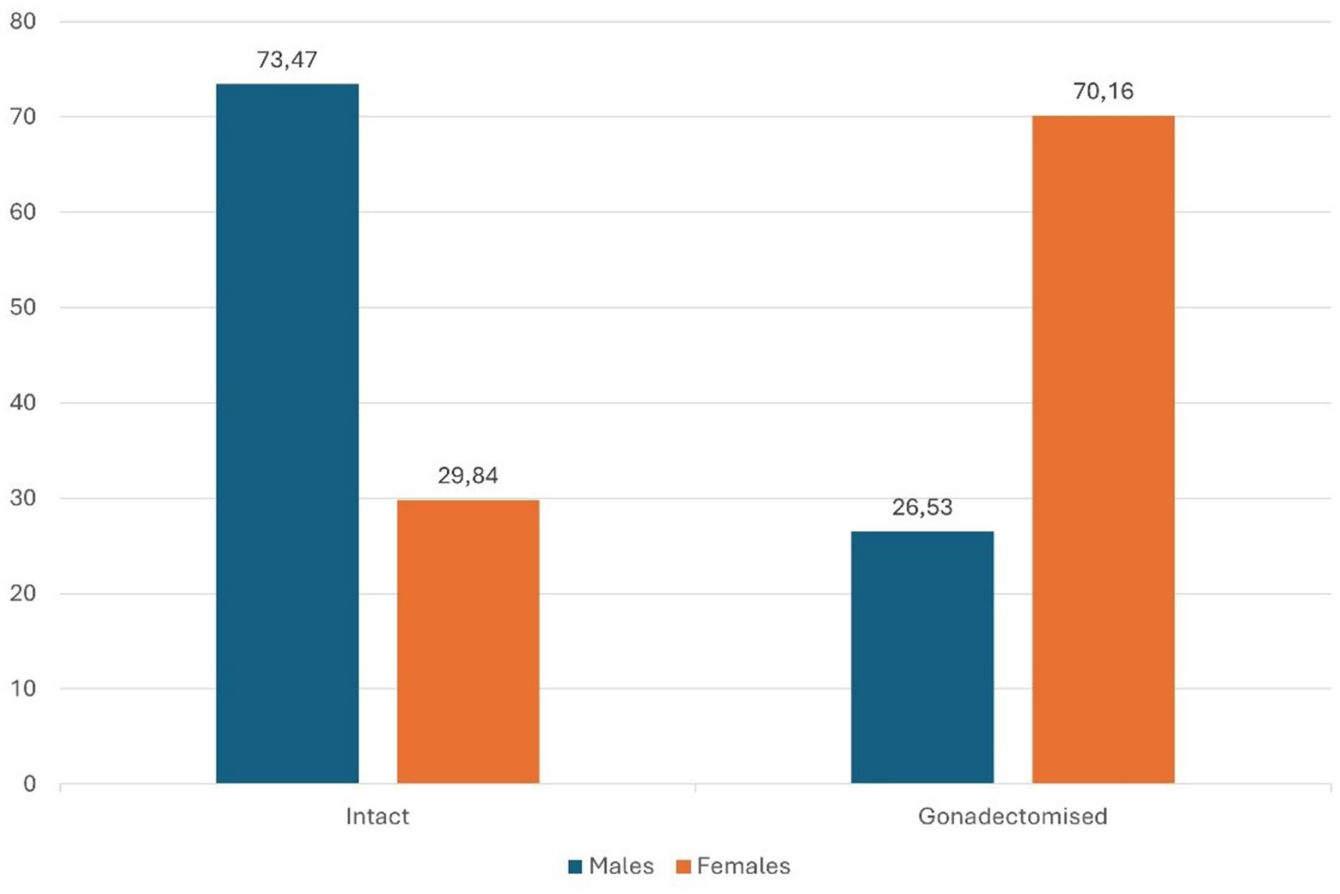
The percentage distribution of intact and gonadectomized subjects categorized by sex.

**Figure 2:**
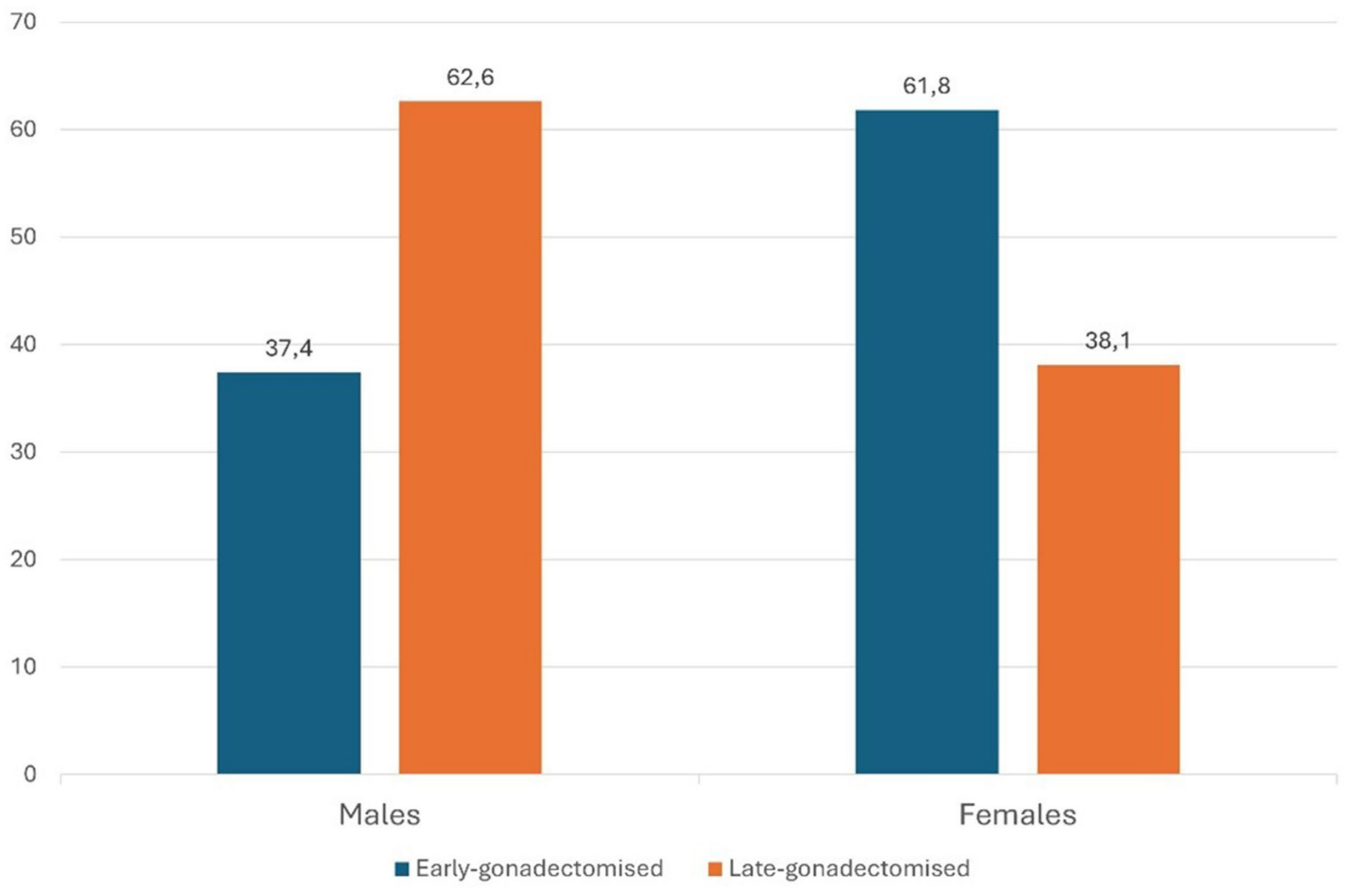
Percentage distribution of males and females gonadectomized according to age at gonadectomy.

The sex distribution and proportions of gonadectomized, IN, EG and LG animals within Group A (Figures 1, 2) are summarized in Table 2.

**Table 2:** Distribution of sex, neuter status, and neuter type in Group A.

Sex	Gonadectomized (%)	Intact (%)	EG (% of gonadectomized)	LG (% of gonadectomized)
Males (57%)	26.53%	73.47%	62.63%	37.37%
Females (43%)	70.16%	29.84%	61.87%	38.13%

IN, intact; EG, early gonadectomized; LG, late gonadectomized.

Statistical analysis revealed that gonadectomy alone was not significantly associated with the risk of IVDH in males, with an OR of 0.975 (95% CI: 0.73–1.29; *p* = 0.863). However, when the data were examined according to age at gonadectomy, EG males showed a significantly higher risk with an OR of 1.92 (95% CI: 1.31–2.81; *p* < 0.05). In contrast, the risk appeared reduced in LG males with an OR of 0.53 (95% CI: 0.35–0.79; *p* < 0.05).

For females, gonadectomy was strongly associated with the risk of IVDH. Gonadectomized females showed a significantly higher risk than IN females, with an OR of 4.996 (95% CI: 3.66–6.81; *p* < 0.0001). The risk was even higher in EG females with an OR of 9.84 (95% CI: 6.71–14.45; *p* < 0.0001). In contrast, the risk was relatively lower in LG females with an OR of 2.77 (95% CI: 1.91–4.02; *p* < 0.0001).

## Discussion

This study evaluated the influence of gonadectomy and age at gonadectomy on the risk of IVDH in French Bulldogs, with the aim of investigate the effect of gonadal sex hormones on intervertebral disc. A significant association was observed between gonadectomy and the risk of developing IVDH, particularly in dogs gonadectomized before 12 months of age, with females showing the strongest relationship. These findings support the hypothesis that sex hormones play a protective effect on the intervertebral disc, especially during skeletal maturation. Overall, the results are consistent with previous studies reporting an increased risk of several orthopedic and spinal disorders following early gonadectomy (22, 26, 28–35).

A high prevalence of neurological conditions has been described in French Bulldogs due to the brachycephalic and chondrodystrophic body conformation resulting from selective breeding, including brain tumors, non-infectious encephalitides and myelopathies such as compressive vertebral malformations, spinal arachnoid diverticula and IVDH (6–10, 36). These findings suggest a strong genetic predisposition, which, combined with environmental factors, may influence the risk of neurological disorders in this breed, such as IVDH (37). To date, IVDH is one of the most common and debilitating neurological disorders in dogs, accounting for almost 4% of admissions to veterinary clinics (11–14, 27, 38). Clinical signs range from lumbar pain to severe neurological deficits and hind limb paralysis in the most advanced cases. The severity of the clinical signs does not directly correlate with the extent of spinal cord compression but may be influenced by spinal cord contusion and other pathophysiological mechanisms (20, 39). Affected dogs may experience a significant reduction in quality of life, necessitating prolonged surgical or medical treatment (13, 14, 40).

The mean age in Group A was approximately 6 years, with minimal variation between sexes and among the IN, EG, and LG subgroups. This value is slightly higher than that reported in previous studies, which described disease onset between three and 5 years of age (1, 11, 21). However, the ages recorded in the present study refer to the time of MRI examination, which may not coincide with the actual onset of clinical signs reported by the owner or documented in the medical record. Because MRI is an expensive and invasive diagnostic technique, it is often performed only when clinical signs worsen or fail to improve, resulting in a delay between onset and diagnosis. This delay may explain the higher mean age at MRI detection and represents an inherent limitation of the retrospective study design.

Regarding the distribution of gonadectomized individuals within the study population, an interesting difference was observed between sexes. Most males in the sample were intact, whereas most females had been gonadectomized. This imbalance likely reflects differences in clinical recommendations and management practices based on sex. Gonadectomy is commonly recommended for females as a preventive measure to reduce the incidence of potentially life-threatening conditions such as pyometra and mammary tumors, and to avoid undesirable hormonally driven phenomena such as pseudopregnancy (29, 41–43). In contrast, gonadectomy in males is generally performed to address behavioral issues or specific medical conditions, and surgery is often avoided in the absence of such indications. Consequently, the higher prevalence gonadectomized females observed in our data aligns with trends reported in other canine populations (44).

The influence of sex hormones on intervertebral disc physiology is supported by several experimental studies. Receptors for estrogen and androgen have been identified in multiple disc cell types, including articular cartilage chondrocytes, annulus fibrosus cells, and nucleus pulposus cells (45–48). Estrogens have been shown to promote cell proliferation and stimulate the synthesis of key extracellular matrix components such as collagen type II, aggrecan, and glycosaminoglycans (47–49). Moreover, they play a protective role by limiting cell senescence and apoptosis, and by preventing calcification of the vertebral endplates (48–53). Testosterone has also been shown to promote matrix protein expression, indicating a potential anabolic role in maintaining disc homeostasis (49). Therefore, the abrupt withdrawal of sex hormones during growth may adversely affect intervertebral disc biomechanics, thereby increasing susceptibility to IVDH. This effect likely arises not only from the absence of hormonal influence itself, but also from complex interactions between sex hormones and cytokine-mediated inflammatory pathways, as well as from potential imbalances between testosterone and estrogen levels (49, 52, 54).

In our study, gonadectomized dogs, particularly EG (those that underwent gonadectomy before 12 months of age), exhibited a higher risk of developing IVDH compared to intact dogs or those LG (gonadectomized after 12 months of age). These findings are consistent with those reported by Dorn and Seath (26), who observed an increased risk of IVDH in Dachshunds gonadectomized before 12 months of age. Similarly, other authors have documented an increased risk of joint and degenerative spinal diseases in Labrador Retrievers, Golden Retrievers, and German Shepherds gonadectomized at an early age (22–25, 28–31). In our study, an important difference in risk between males and females in relation to reproductive status was found. While gonadectomy itself was not associated with an increased risk of IVDH in males (OR = 0.975), a statistically significant finding emerged regarding age at gonadectomy. Males who were EG (gonadectomized before 12 months of age) were almost twice as likely to develop IVDH as intact males (OR = 1.92), whereas LG (those gonadectomized later) showed a reduced risk (OR = 0.53). In contrast, presentation of IVDH appeared to be influenced more significantly by reproductive status in females. Gonadectomized females were found to be at a significantly higher risk of developing IVDH than intact females (OR = 4.996), with those EG (gonadectomized at an early age) being almost 10 times more likely to develop the condition (OR = 9.84). Late-gonadectomized (LG) females were also at higher risk than intact females, albeit with a lower OR (2.77).

The irreversible loss of gonadal hormones after gonadectomy results in an alteration of the hypothalamic–pituitary–gonadal (HPG) axis and a chronic increase in LH and FSH concentrations (44, 55). This endocrine imbalance could negatively affect various tissues, including connective and cartilage tissue, thereby contributing to disc degeneration. Furthermore, early gonadectomy can delay the closure of growth plates, affect skeletal morphology and increase the risk of orthopedic problems (56, 57). Large-scale retrospective studies have shown that early gonadectomy is associated with a higher incidence of orthopedic conditions, such as cranial cruciate ligament rupture and hip dysplasia. Guide dogs that were gonadectomized before 7 months of age were more than twice as likely to develop orthopedic problems as those that were gonadectomized later (44, 58).

Our findings confirm the hypothesis that gonadectomy, particularly before skeletal maturity is reached, may increase the risk of IVDH in French Bulldogs. These findings highlight the need for a breed-specific approach to gonadectomy, based on an accurate assessment of the risks and benefits. Current recommendations should consider breed, size and age of the dog, as well as concurrent disease and its genetic predisposition to specific diseases. However, the potential long-term adverse effects must be balanced against these benefits, particularly when gonadectomy is performed at a very early age. While some studies have supported the safety of pediatric gonadectomy (6–16 weeks) (44, 59, 60), others have highlighted an increased risk of orthopedic and musculoskeletal problems in dogs gonadectomized at an early age (26, 28–31).

The reduction in genetic diversity caused by the systematic gonadectomy of unselected individuals may contribute to the spread of hereditary diseases. Although gonadectomy limits the reproduction of individuals with known genetic defects, it can also encourage the fixation of harmful recessive alleles that have not yet been identified, particularly in breeds with a small effective population, such as French Bulldogs. While it is widely recognized that gonadectomy brings significant benefits to the overall animal population, it also involves advantages and potential risks for the individual. Due to the complex interaction between multiple etiological factors influencing the development of various medical and behavioral disorders, it is impossible to predict the outcome of gonadectomy for each animal accurately. Therefore, the decision to neuter a pet should be made after carefully considering the patient’s specific conditions, the owner’s values and goals, and the available epidemiological data on risks and benefits.

## Conclusion

Results showed a significant difference in the association between gonadectomy and the risk of IVDH in French Bulldogs. No significant increase in risk has been found when comparing all gonadectomized males and intact males. Nevertheless, early gonadectomized males were two times more likely to develop IVDH when compared to intact males. In females, the association between gonadectomy and IVDH is much stronger than in males, with an approximately 5-fold increased odds. In addition, early gonadectomized females had an almost 10-fold increased odds compared with intact females. These data emphasize the crucial importance of age at gonadectomy as a determining factor in the risk of IVDH. A personalized approach to the reproductive management of French Bulldogs and, more broadly, chondrodystrophic breeds is recommended. The decision to neuter, and especially the timing of gonadectomy, should be based on an integrated risk assessment that considers individual factors, genetic predispositions, sex, and clinical and management objectives. Ultimately, our findings underscore the necessity for additional prospective, multicenter, controlled studies to investigate the pathogenic mechanisms involved and establish evidence-based guidelines for more targeted and informed clinical practices that priorities the long-term welfare of the animal.

## Data Availability

The original contributions presented in the study are included in the article/supplementary material, further inquiries can be directed to the corresponding authors.
